# RecQ Helicase Somatic Alterations in Cancer

**DOI:** 10.3389/fmolb.2022.887758

**Published:** 2022-06-15

**Authors:** Megha K. Thakkar, Jamie Lee, Stefan Meyer, Vivian Y. Chang

**Affiliations:** ^1^ Department of Pediatrics, Division of Pediatric Hematology-Oncology, University of California, Los Angeles, Los Angeles, CA, United States; ^2^ Division of Cancer Studies, University of Manchester, Manchester, United Kingdom; ^3^ Department of Pediatric Hematology Oncology, Royal Manchester Children’s Hospital and Christie NHS Foundation Trust, Manchester, United Kingdom; ^4^ Childrens Discovery and Innovation Institute, UCLA, Los Angeles, CA, United States; ^5^ Jonsson Comprehensive Cancer Center, UCLA, Los Angeles, CA, United States

**Keywords:** RecQ helicases, RECQ1, BLM, WRN, Recql4, Recql5, cancer

## Abstract

Named the “caretakers” of the genome, RecQ helicases function in several pathways to maintain genomic stability and repair DNA. This highly conserved family of enzymes consist of five different proteins in humans: RECQL1, BLM, WRN, RECQL4, and RECQL5. Biallelic germline mutations in *BLM*, *WRN*, and *RECQL4* have been linked to rare cancer-predisposing syndromes. Emerging research has also implicated somatic alterations in RecQ helicases in a variety of cancers, including hematological malignancies, breast cancer, osteosarcoma, amongst others. These alterations in RecQ helicases, particularly overexpression, may lead to increased resistance of cancer cells to conventional chemotherapy. Downregulation of these proteins may allow for increased sensitivity to chemotherapy, and, therefore, may be important therapeutic targets. Here we provide a comprehensive review of our current understanding of the role of RecQ DNA helicases in cancer and discuss the potential therapeutic opportunities in targeting these helicases.

## 1 Introduction

The highly conserved RecQ family of proteins play fundamental roles in the maintenance of genomic stability (Hickson, 2003; Croteau et al., 2014). Impaired function in these RecQ proteins widely impacts health and has been associated with cancer and aging (Ellis et al., 1995; Yu et al., 1996; Kitao et al., 1999; Kyng et al., 2003; Orren, 2006; Kudlow et al., 2007). There are at least five human RecQ helicase proteins, RECQL1, BLM, WRN, RECQL4, and RECQL5. Biallelic, loss-of-function germline pathogenic variants in three of them, *BLM*, *WRN*, and *RECQL4*, have been linked to rare cancer-predisposing syndromes. More recently, mutations in *RECQL1* have been identified with the novel genome instability disorder called RECON (RECql ONe) syndrome though cancers have not yet been described in these patients (Abu-Libdeh et al., 2022).

**FIGURE 1:**
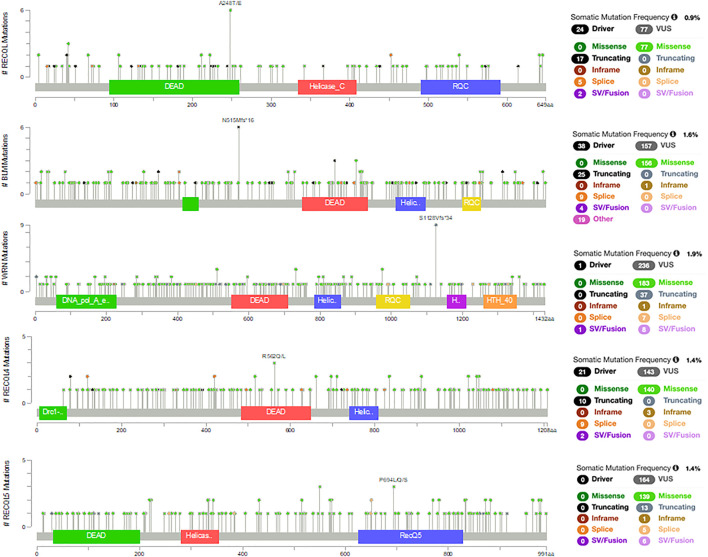
Somatic mutations in RecQ helicases from the PanCancer Atlas with 10,967 samples of different cancer types (Cerami et al., 2012; Gao et al., 2013).

The PanCancer Atlas with 10,967 samples of different cancer types shows that these RecQ helicase genes are somatically mutated in 0.9–1.9% of cases (Figure 1) (Cerami et al., 2012; Gao et al., 2013). These missense, truncating, inframe, splice, and structural variants/fusions have been identified across these genes with no clear somatic hot spots. Of note, RECQL5 has no known driver mutations, although there are 164 variants of uncertain significance. Despite the relative paucity of somatic mutations, the RecQ helicase family has significant roles in cancer. This review aims to provide a summary of the RecQ protein family structure and function, and the various roles in cancer, with a focus on somatic alterations that drive tumorigenesis and potential therapeutic opportunities through small molecules or a synthetic lethal approach (Table 1).

**TABLE 1:** Summary of germline and somatic alterations in RecQ helicases with potential anti-cancer therapeutic opportunities.

	RECQL1	BLM	WRN	RECQL4	RECQL5
Germline alterations		Bloom	Werner	Rothmund-Thompson, RAPADILINO, Baller-Gerold	
Somatic alterations	-Glioblastoma	-Breast carcinoma	-Colorectal carcinoma	-Osteosarcoma	-Breast carcinoma
	-Ovarian carcinoma	-Lung adenocarcinoma	-Gastric carcinoma	-Non-melanoma skin cancers	-Gastric carcinoma
	-Head and neck squamous cell carcinoma	-Prostate carcinoma	-Breast carcinoma	-Lymphomas	-Colorectal carcinoma
	-Hepatocellular carcinoma	-Basal cell carcinoma	-Cervical carcinoma	-Breast carcinoma	-Urothelial bladder carcinoma
	-Colorectal carcinoma	-Colorectal carcinoma	-Multiple myeloma	-Prostate carcinoma	-Acute myeloid leukemia
	-Breast carcinoma	-Gastric carcinoma		-Cervical carcinoma	-Diffuse large B-cell lymphoma
	-Multiple myeloma	-Acute myeloid leukemia		-Acute myeloid leukemia	-Chronic lymphocytic leukemia
	-Acute myeloid leukemia			-Multiple myeloma	-Non-small cell lung carcinoma
	-Pancreatic carcinoma			-Esophageal squamous cell carcinoma	
				-Ovarian carcinoma	
				-Gastric carcinoma	
				-Hepatocellular carcinoma	
				-Glioblastoma	
Therapeutic approaches	-Inhibition of RECQL1 plus inhibition of PARP1/TOP1	-Small-molecule inhibitors -- ML216 and its derivatives	-Inhibition of WRN plus cleavage of TA-dinucleotide repeats by MUS81 nuclease	-Alter RECQL4 expression to affect stem cells	-Small-molecule inhibitors -- 1,3,4-oxadiazole derivatives to inhibit homologous recombinatorial repair
	-siRNA silencing of RECQL1	-Alteration of c-Myc pathways	-Inhibition of WRN plus target CHK1-p38-MAPK pathway	-Compounds similar to antibiotic heliquinomycin	-Target RECQL5 to enhance sensitivity to camptothecins
	-Combination of DNA methyltransferase inhibitors (DNMTi), PARPi, and inhibitors against RECQL1 helicase		-Small-molecule inhibitors -- NSC 19630 (1-(propoxymethyl)-maleimide) and its derivative NSC 617145, NCGC00029283-03 and NCGC00063279-03		-Inhibition of PI3K/Akt signaling pathway

### 1.1 RecQ Protein Structure

Sequence analyses have identified three domains conserved across bacteria and eukaryotes, the helicase, RecQ carboxy-terminal (RQC), and helicase and RNase D C-terminal (HRDC) domains that interact with many proteins involved in DNA replication, recombination, and repair (Morozov et al., 1997). The core functional unit is comprised of the ATPase-dependent RecQ helicase domain and the RQC. The helicase domain has a single strand DNA sensor element and a DNA-binding region that utilizes ATP hydrolysis to catalyze unwinding of complementary of DNA in a 3′ to 5’ direction. The RQC domain is less conserved across the RecQ family and is only partially present in RECQL4 and RECQL5. It is thought to regulate contact with DNA and be involved in oligomerization status of the helicase (Soultanas and Wigley, 2001; Singleton et al., 2007; Pike et al., 2009; Lucic et al., 2011). The HRDC domain is the least conserved region of the RecQ helicase family, being present only in BLM and WRN, as well as bacterial and yeast homologues (Morozov et al., 1997). It mediates DNA binding activity of the helicase and RQC core but appears to be dispensable for enzyme function both *in vitro* and *in vivo* (Mullen et al., 2000; Mullen et al., 2001; Bernstein and Keck, 2003; Janscak et al., 2003).

WRN is unique amongst the RecQ family proteins because in addition to the helicase domain, it also has 3′ to 5’ exonuclease domain (Huang et al., 1998; Choi et al., 2007). This domain is located in the N terminus and becomes activated upon binding of DNA, independently of its helicase domain (Choi et al., 2007). WRN helicase interacts with DNA2 nuclease to degrade reversed replication forks and stimulate replication restart, while the exonuclease activity may play in a role in protection of the stalled fork (Thangavel et al., 2015; Datta et al., 2021).

Remarkably, the R4ZBD domain of the RECQL4 structure is different from RQC domains in other RecQ helicases in that it contains a new C-terminal domain (Kaiser et al., 2017). This unique domain consists of a zinc-binding motif and characteristic winged-helix domains that do not act in expected DNA binding or helicase activities. Rather, it may be involved in protein stability, and mutations may lead to cancers and specific RECQL4-associated syndromes (Kaiser et al., 2017).

### 1.2 RecQ Functions in DNA Repair Pathways

DNA is constantly being damaged by a variety of endogenous and exogenous factors, which then trigger different cellular pathways to repair damaged DNA lesions (Lindahl, 1993; Friedberg et al., 2006). When DNA damage remains unresolved, this results in genomic instability with accumulation of mutations and chromosomal rearrangements, which are then associated with cell death, accelerated aging, and cancer (White and Vijg, 2016; Tubbs and Nussenzweig, 2017; d'Adda di Fagagna, 2008). Each RecQ family protein is involved in multiple aspects of DNA repair, both shared and unique.

### 1.3 Homologous Recombination

Homologous recombination (HR) repairs double-strand breaks (DSB) using a homologous DNA template, primarily restricted to the S and G2 phases of cell cycle. HR also plays an important role in DNA replication and telomere maintenance. Alterations to the HR pathway are known to cause genomic instability and drive cancer, as well as affect sensitivity of cancers to chemotherapy (Thomas and Capecchi, 1987; Scully et al., 1997; Moynahan et al., 1999; Canto et al., 2019).

BLM is involved in HR through its interactions with EXO1 and DNA2 (Nimonkar et al., 2008; Nimonkar et al., 2011; Sturzenegger et al., 2014), resecting DSB and generating 3′ single-stranded DNA that can then be bound by replication protein A (RPA) and RAD51 (Gravel et al., 2008; Nimonkar et al., 2008; Nimonkar et al., 2011). RAD51 searches for a homologous DNA template, leads strand invasion and creates a “D-loop” structure where two strands of double-stranded DNA are separated. Once DNA is resynthesized, BLM then processes these two strands of double-stranded DNA (dsDNA), Holliday junctions, and restores separate DNA duplexes, in a complex with Topoisomerase Iiα and RMI1/2 (Wu and Hickson, 2003; Singh et al., 2008). BLM also builds up at stalled replication forks and interacts with FANCM and FANCC to dissolve double Holliday junctions (Davalos and Campisi, 2003; Wu and Hickson, 2003; Singh et al., 2008; Moder et al., 2017). This process normally limits genetic exchange from occurring between homologous sequences during HR, which is the basis for the increased sister chromatid exchange observed in cells from patients with Bloom syndrome (Wu and Hickson, 2003; Wang et al., 2015a). The interaction between TopBP1 and BLM has been suggested to play a role in suppressing sister chromatid exchange by maintaining BLM in the S phase cycle (Singh et al., 2008). Additionally, BLM and RECQL5 both play an anti-recombinase role by disrupting RAD51 nucleoprotein filaments from resected DNA and preventing RAD51-mediated D-loop formation thereby regulating HR (Hu et al., 2007; Patel et al., 2017). RECQL5 inhibits the exonuclease activity of MRE11, part of the MRE11-RAD50-NBS1 (MRN) complex. The MRN complex senses DNA DSBs and recruits RECQL5 to sites needing DNA repair (Zheng et al., 2009) This suggests that RECQL5 potentially controls errors in HR, thereby acting as a crucial tumor suppressor (Hu et al., 2007; Zheng et al., 2009). In addition to promoting NHEJ, RECQL4 is also involved in homologous recombination (HR)-dependent DNA DSB repair. This occurs when RECQL4 interactions initiate 5’ DNA end resection (Lu et al., 2016; Lu et al., 2017).

### 1.4 Nonhomologous End-Joining

Nonhomologous end-joining (NHEJ) is a template-independent DSB repair process that is more error-prone than homologous recombination but is active across phases of the cell cycle. Cells that lack various components of the NHEJ pathway display increased genomic rearrangements (Karanjawala et al., 1999). NHEJ itself can also induce genomic arrangements by ligating two junctions of DSBs that are not compatible, resulting in insertions or deletions (Rothkamm et al., 2001; Ghezraoui et al., 2014). Therefore, NHEJ has a dual role in suppressing and promoting cancer (Sishc and Davis, 2017).

The five human RecQ helicases are involved in DSB repair. WRN promotes canonical NHEJ by interacting with Ku, the loading protein that recruits other NHEJ proteins, which in turn stimulates WRN exonuclease activity (Li and Comai, 2000; Karmakar et al., 2002a; Kim et al., 2020). DNA-PKcs, another important protein involved in NHEJ, phosphorylates WRN, inhibiting helicase activity and mediating the re-localization of WRN to the nucleolus (Karmakar et al., 2002b; Kusumoto-Matsuo et al., 2010; Kusumoto-Matsuo et al., 2014). BLM helicase has recently been shown to be required for recruitment of NHEJ component, XRCC4, to chromatin in the G1 phase and also negatively regulate NHEJ during S-phase (Tripathi et al., 2018). Both RECQL1 and RECQL4 interact with the Ku70/80 subunit of the DNA-PK complex. This interaction is important to modulate DNA double-strand break repair via nonhomologous end-joining (NHEJ) (Parvathaneni et al., 2013; Shamanna et al., 2014; Lu et al., 2017).

### 1.5 Base Excision Repair

Base excision repair (BER) is the predominant pathway utilized to repair endogenous DNA damage, such as deaminations, alkylations, oxidative damage, and abasic single base damage (Mitra et al., 1997; Zharkov, 2008; Dianov and Hubscher, 2013). BER is initiated by DNA glycosylases, which demonstrate specificity for damaged DNA bases and conduct specific mechanisms to excise these lesions. The next step of the BER pathway consists of recognition of the AP site by AP endonuclease 1 (APE-1), which cleaves the abasic site, then eventually for DNA polymerase ß and DNA ligase to complete BER via repair and replacement of a single damaged DNA nucleotide (Carter and Parsons, 2016).

Key protein interactions overlap between SSB repair and the BER pathway (Popuri et al., 2013). BLM and WRN interact with many proteins involved in BER and single-strand break repair (SSBR) pathways. WRN interacts with APE-1. WRN and BLM stimulate several BER proteins including DNA polymerase β, FEN-1, and NEIL1 (Brosh et al., 2001; Harrigan et al., 2003; Sharma et al., 2004). RECQL4 interacts with several proteins to promote BER, including FEN1, APE1, and PARP1, where these protein interactions are essential to conduct oxidative damage (Luong and Bernstein, 2021). RECQL5 interacts with FEN1 and PCNA proteins, involved in BER (Popuri et al., 2013).

### 1.6 Telomere Maintenance

Telomeres, which protect the ends of chromosomes, have an important function in maintaining genomic integrity. Telomere length decreases with age as a result of normal processes that lead to cellular senescence and organismal aging. DNA damage, repeated cell division, and variants in the telomere protective complex can cause shortening and dysfunction of telomeres, leading to cell death, senescence, or additional genomic instability (Bejarano et al., 2019; McNally et al., 2019). Interestingly, both shorter and longer telomere lengths have been associated with cancer predisposition and cancer-associated mortality (Telomeres Mendelian Randomization et al., 2017; McNally et al., 2019; Samavat et al., 2019; Protsenko et al., 2020; Schmutz et al., 2020).

Patients with Werner Syndrome have features of accelerated aging and cells from these patients display shortened telomere lengths (Ouellette et al., 2000; Wyllie et al., 2000; Chang et al., 2004; Crabbe et al., 2007). Consistent with this aging phenotype, it has been demonstrated that the WRN helicase is necessary for replication of G-rich telomeric DNA (Crabbe et al., 2004). WRN and BLM are also involved in the alternative lengthening of telomeres (ALT) pathway that is independent of telomerase, by localizing to telomeric DNA and using HR to lengthen telomeres (Bryan et al., 1995; Yankiwski et al., 2000; Johnson et al., 2001). Similar to WRN and BLM, RECQL4 has also been linked with telomere maintenance by supporting telomeric D-loop resolution through its interaction with shelterin proteins as well as through WRN stimulation (Ghosh et al., 2012; Kaiser et al., 2017).

### 1.7 Replication Stress

During DNA replication, dsDNA that are separated into two branching single strands form a replication fork. Stalled replication forks lead to genomic instability and chromosomal rearrangements which are associated with cancer. RecQ helicases interact with replication forks in several ways by unwinding DNA, assisting with branch migration, and strand annealing (Bachrati and Hickson, 2008; Bohr, 2008; Vindigni et al., 2010). BLM can promote fork reversal and restart, and suppress firing (Davies et al., 2007; Machwe et al., 2011a; Machwe et al., 2011b; Bugreev et al., 2011). WRN is also required after replication arrest via MUS81 endonuclease activity (Franchitto et al., 2008). RECQL1 is involved in reverse branch migration, while RECQL5 disrupts RAD51 filaments on stalled replication forks by also interacting with endonuclease MUS81 (Urban et al., 2016; Di Marco et al., 2017; Chappidi et al., 2020).

## 2 Overview of DNA RecQ Helicases in Cancer

### 2.1 RECQL1

#### 2.1.1 Germline Variants in *RECQL1*

Missense biallelic mutations in RECQL1 gene are associated with the novel RECql One (RECON) syndrome. Remarkably, although individuals with RECON syndrome display overlapping features with the other RecQ-associated genetic disorders, the overall clinical phenotype is distinct. The characteristic features include progeroid facies, skin photosensitivity, xeroderma, pinched nose, and elongated thumbs (Abu-Libdeh et al., 2022). Studies recently demonstrated that these mutations affect the ability to repair DNA damage and perform successful DNA replication once exposed to topoisomerase inhibitors. More studies are needed to determine if there is an association with cancer development (Abu-Libdeh et al., 2022).

There are conflicting studies investigating germline variants in *RECQL1* as a cancer susceptibility gene. Germline missense, truncating, and splice site variants in *RECQL1* have been enriched in participants with breast cancer in five different ethnic groups (Cybulski et al., 2015; Sun et al., 2015; Kwong et al., 2016; Sun et al., 2017; Tervasmaki et al., 2018). However, there have been a few subsequent studies with conflicting evidence, such that *RECQL1* appears to be a moderate breast cancer risk gene that needs further study (Bogdanova et al., 2017; Li et al., 2018a; Nguyen-Dumont et al., 2018).

One study evaluated 13 single nucleotide polymorphisms (SNPs) in DNA repair genes, including *RECQL1*, in patients with pancreatic adenocarcinoma (Li et al., 2006). A polymorphism in *RECQL1* A159C had the strongest effect on survival and strongly interacted with other genotypes in *RAD54L*, *XRCC1*, and *ATM*. Although the functional impact of this polymorphism is unknown, it is possible that deficient *RECQL1* may lead to an aggressive tumor subtype through increased genomic instability (Li et al., 2006).

### 2.2 RECQL1 and Cancer

*RECQL1* is highly expressed in proliferating cells and has been shown to be upregulated in multiple cancer cell lines, as part of a common cancer signature identified through cancer microarray data (Kawabe et al., 2000; Xu et al., 2007; Futami et al., 2008a). Overexpression of *RECQL1* has been found in glioblastoma, ovarian cancer, and head and neck squamous cell carcinoma (Arai et al., 2011; Mendoza-Maldonado et al., 2011; Sharma, 2011).

Hypopharyngeal carcinomas, an aggressive subtype of head and neck squamous cell carcinoma, highly express RECQL1 and WRN proteins (Arai et al., 2011). Cell lines and clinical samples of hypopharyngeal carcinoma showed increased ɣH2AX, a marker of proliferation and activated DNA damage response. Tumor cell growth was significantly inhibited by *RECQL1*-or *WRN*-silencing by siRNA. Furthermore, *in vivo* combination treatment with cisplatin and siRNA silencing of *RECQL1* or *WRN* led to increased DNA damage, apoptosis, and mitotic catastrophe.

High expression of *RECQL1* has been significantly associated with poor overall survival in breast cancer patients (Gyorffy et al., 2010). In 595 breast cancer patient samples found in the Cancer Genome Atlas (TCGA) database, *RECQL1* mRNA expression was correlated with several other genes, including *CDK6*, *CENPA*, and *TGFBI*, suggesting that *RECQL1* may regulate expression of other critical genes involved in cancer cell migration, invasion, and metastasis (Li et al., 2014). Zhu et al. investigated a large sample size of breast cancer patients with various tumor characteristics. They found that increased expression of *RECQL1* was significantly associated with reduced relapse-free survival and post-progression survival in all breast cancers but improved overall survival in patients with basal-like breast cancer and mutant-p53-type breast cancer (Wang et al., 2009).

RECQL1 protein levels are elevated in ovarian cancer and correlate with histological type and high proliferative potential (Sanada et al., 2013). There was, however, no association between RECQL1 expression and overall survival. When ten ovarian cancer cell lines of various histologic subtypes were subject to siRNA silencing of *RECQL1*, cancer cells decreased in number through mitotic cell death, compared to two normal cell lines. These results suggest a potential anticancer effect of *RECQL1* silencing (Futami et al., 2008b; Sanada et al., 2013).

Additionally, RECQL1 protein expression was identified in the majority of hepatocellular carcinoma (HCC) samples by immunohistochemistry and correlated with histological grade, portal vein invasion, and tumor size >2 cm (Futami et al., 2010). Silencing of *RECQL1* using siRNA induced mitotic catastrophe, leading to cell death in various HCC cell lines and demonstrating anticancer activity *in vivo*. This has been proposed as a potential therapeutic approach against HCC.

*RECQL1*, *WRN*, and *RECQL5* mRNA expression have been found to be lower in primary colorectal carcinoma (CRC) samples compared to normal colonic mucosa, while *BLM* and *RECQL4* mRNA levels are increased (Lao et al., 2013). When evaluating molecular subtypes of CRC, increased *BLM* expression was the only RecQ gene correlated with CpG island methylator phenotype (CIMP) status. CRC with microsatellite instability (MSI) had significantly lower *RECQL1* and *RECQL5* expression compared to normal colonic tissue. There were, however, no differences in protein expression of any of the RecQ helicases by immunohistochemistry between localized disease compared to advanced stage disease (Lao et al., 2013).

The role of RECQL1 in replication stress is highlighted in multiple myeloma. In a study by Viziteu et al. focusing on multiple myeloma cells, *RECQL1* overexpression was significantly higher in primary myeloma cells from newly diagnosed patients compared to normal bone marrow plasma cells (Viziteu et al., 2016). Additionally, overexpression of *RECQL1* was correlated with poorer prognosis, while depletion led to apoptosis and arrest of cell growth in multiple myeloma cells (MMC) (Viziteu et al., 2017). Forced expression of *RECQL1* in human myeloma cell lines correlated with an increased resistance to melphalan and bortezomib-induced cell death. Additionally, it was found that *RECQL1* depletion induced cytotoxicity in MMCs by PARP inhibitors. This data demonstrates the crucial role of RECQL1 in not only protecting multiple myeloma cells against DNA damage and replicative stress but also against chemotherapeutic agents (Viziteu et al., 2016). As DNA methyltransferase (DNMT) inhibitors can lead to decreased expression of *RECQL1*, MMCs can potentially be targeted using combination therapy of DNMT inhibitors and chemotherapy and/or PARP inhibitors (Viziteu et al., 2017).

### 2.3 BLM

#### 2.3.1 Germline Variants in *BLM* Cause Bloom Syndrome

Bloom Syndrome (BSyn) is an autosomal recessive disease caused by variants in the *BLM* gene, located at the 15q26.1 locus (German et al., 1994). Patients with BSyn typically present with short stature, immunodeficiency, skin photosensitivity, and susceptibility to cancer (Mojumdar, 2020). Chromosomes in cells from patients with BSyn display increased frequency of sister chromatid exchanges secondary to the role of BLM in homologous recombination described above. This is thought to be the underlying mechanism for their increased risk of cancer (Ababou, 2021). They develop a wide spectrum of cancer types, with hematologic and gastrointestinal malignancies being most frequent, at younger ages than the general population.

Heterozygous carriers of *BLM* variants have not been extensively studied but there are a few studies showing mixed results with regard to cancer risk (Cleary et al., 2003; Baris et al., 2007; Antczak et al., 2013; Laitman et al., 2016; Schayek et al., 2017). One study examined patients with metastatic prostate cancer who underwent germline genetic testing and found that truncating *BLM* variants occurred at a higher frequency in patients with metastatic prostate cancer than control populations (Ledet et al., 2020). Several studies in patients with colon cancer have shown an enrichment of deleterious *BLM* variants (Gruber et al., 2002; de Voer et al., 2015). An elegant study examining gene-environment links demonstrated that germline *BLM* variants increase susceptibility to asbestos-related carcinogenesis, leading to mesothelioma, a link first identified in patients with mesothelioma and confirmed in a mouse model (Bononi et al., 2020). Smaller studies trying to link germline variants in BLM and breast cancer have yielded mixed results (Thompson et al., 2012; Kluzniak et al., 2019).

Case-control studies have shown associations between polymorphisms in *BLM* and breast cancer (Wirtenberger et al., 2006; Broberg et al., 2009; Ding et al., 2009; Sassi et al., 2013). One study investigated polymorphisms in *WRN*, *RMI1*, and *BLM* in a large population-based case-control study of colorectal cancer patients and controls and found an association of *BLM* P868L with rectal cancer risk (Frank et al., 2010). One haplotype overlapping with *BLM* was shown to be significantly associated with prostate cancer susceptibility (Wang et al., 2015b). Further studies are needed to better understand these polymorphisms in *BLM* and their influence on cancer risk and prognosis.

### 2.4 BLM and Cancer

Studies have shown that overexpression of *BLM*, both mRNA and protein, has significant prognostic value in breast cancer (Arora et al., 2015; Zhu et al., 2018). In the previously mentioned study by Zhu et al., increased expression of *BLM* was associated with reduced distant metastasis-free survival in all patients (Zhu et al., 2018).

An integrated genomic approach analyzing two cohorts of triple-negative breast cancers resistant to cisplatin therapy, showed that *BLM* had increased DNA copy number and gene expression in cases that responded to cisplatin chemotherapy (Birkbak et al., 2018). Using lentiviral overexpression of *BLM* in cell lines, it has been shown that increased *BLM* expression promotes increased sensitivity to cisplatin. The mechanism of this finding seems to be through increased DNA damage seen in *BLM* overexpressing cells, potentially due to predominating anti-HR effects of BLM at high levels (Birkbak et al., 2018).

One recent study on lung adenocarcinoma validated a cancer stem cell-related biomarker by an mRNA stemness index (mRNAsi) that was significantly higher in patients with lung cancer than controls (Zhao et al., 2020). Lung cancer patients with higher mRNAsi also had higher stage cancers and worse overall survival. *BLM* was found to be differentially expressed amongst lung cancer patients and low expression of *BLM* was significantly correlated with better overall survival (Zhao et al., 2020). A second study profiling the mutational landscape of lung cancer identified *BLM* as being one of nine recurrently mutated genes (Zhou et al., 2020). Interestingly, mutations in *BLM* were found in higher frequently in male patients compared to female patients. A subset of patients with *BLM* mutations had no clinically actionable targets such as *KRAS, ERBB2, MET, RET, BRAF* and *PIK3CA*, leaving gaps for future novel therapies (Zhou et al., 2020).

*BLM* mRNA and protein expression are upregulated in prostate cancer cells compared to controls (Qian et al., 2017). Inhibition of *BLM in vitro* reduces cell proliferation and increases apoptosis, while having no effect on prostate cancer cell migration and invasion. One study investigated the role of *BLM* in prostate cancer progression (Chen et al., 2019). Using CRISPR/Cas9, they showed that *BLM* deletion in prostate cancer cells inhibits cell proliferation by downregulating pAKT and pRAS, which leads to increased reactive oxygen species production (Chen et al., 2019).

When mice with a hypomorphic Blm allele (*Blm* ^*tm3Brd*^) were crossed with a *Ptch1* ^*+/−*^ mouse model of basal cell nevus syndrome, there was significantly increased formation of basal cell carcinoma (BCC) and rhabdomyosarcoma (RMS) (Aszterbaum et al., 1999; Luo et al., 2000; Davari et al., 2010). Although tumor histology and DNA copy number were not significantly different, mice with *Blm* deficiency in the background of *Ptch* heterozygosity had decreased survival. Similarly, mutant Blm increases intestinal tumor formation in Apc^+/min^ mice without affecting tumor histology or chromosomal aberrations (Goss et al., 2002; Traverso et al., 2003; Snijders et al., 2008). These studies therefore demonstrated that *BLM* deficiency increases tumor formation in a tissue-specific pattern.

Patients with BSyn have presented with numerous colonic adenomas, similar to attenuated familial adenomatous polyposis (Lowy et al., 2001). Some adenomas from patients with BSyn harbor somatic mutations in *APC* and display microsatellite instability (Calin et al., 2000; Lowy et al., 2001). Somatic frameshift mutations in *BLM* have been identified in colon cancers and gastric cancers with high microsatellite instability (Calin et al., 2000; Calin et al., 2001). Additionally, it has been shown that *BLM* expression, by both mRNA and protein levels, is increased in colon cancer samples, along with *RECQL4*, while *RECQL1* and *RECQL5* expression are significantly decreased (Lao et al., 2013).

The majority of colon cancer samples and cell lines have overexpression of c-Myc at the RNA and protein levels (Finley et al., 1989; He et al., 1998). Significant overlap between tumors that over-express c-Myc and show loss of *BLM* has been observed (Chandra et al., 2013).

### 2.5 WRN

#### 2.5.1 Germline Variants in *WRN* Cause Werner Syndrome

Werner Syndrome is an autosomal recessive disorder caused by mutations in the *WRN* gene, located on chromosome 8p11. It is associated with short stature, skin atrophy, and premature aging comorbidities, including premature atherosclerosis, stroke, myocardial infarction, cataracts, diabetes mellitus, and osteoporosis (Lauper et al., 2013). These patients have an increased risk of cancers, particularly sarcomas, thyroid carcinoma, meningioma, and hematologic malignancies (Lauper et al., 2013).

A case-control study looking at single-nucleotide polymorphisms in *WRN* identified *WRN* c.4330T > C to be associated with increased susceptibility to esophageal carcinoma (Li et al., 2012). There have been additional small reports of p.C1367R being associated with increased risk of breast cancer but also protective against non-Hodgkin lymphoma and soft tissue sarcoma (Shen et al., 2006; Nakayama et al., 2008; Zins et al., 2015).

*WRN* Leu1074Phe was evaluated for prostate cancer risk and found to potentially increase risk in patients younger than 72 years of age (Wang et al., 2011a). This same polymorphism was associated with an increased risk of breast cancer, earlier age at menarche, as well as an association with onset of cardiovascular disease (Castro et al., 2000; Wang et al., 2009).

### 2.6 WRN and Cancer

Loss of heterozygosity involving the *WRN* loci at chromosome 8p11.2-p12 occurs frequently in many different cancers, pointing to its role as a tumor suppressor gene (Chughtai et al., 1999; Armes et al., 2004). It has been shown that the *WRN* promoter is commonly hypermethylated, thereby leading to silencing of *WRN* expression, in different human cancer cell lines (Agrelo et al., 2006). Indeed, in colorectal cancer, one study demonstrated that epigenetic silencing of *WRN* was associated with improved survival in patients treated with a topoisomerase inhibitor, while another study was not able to validate these findings (Agrelo et al., 2006; Bosch et al., 2016). Gastric cancer samples have also been shown to have *WRN* promoter methylation. Patients with *WRN* methylation and methylation of heparan sulfate 6-O-endosulfatase (*SULF2*) had increased sensitivity to chemotherapy irinotecan but no significant clinical or pathologic correlations were identified (Wang et al., 2013).

Colon cancer samples with somatic variants in *WRN* were more likely to be from right-sided cancers and were associated with increased tumor mutation burden and microsatellite instability (MSI) (Zimmer et al., 2020). *WRN* has been found to have a synthetic lethal interaction with MSI, making it an interesting therapeutic target to be discussed further below (Kategaya et al., 2019; Picco et al., 2021). In addition, *WRN*-mutated colon cancer has a characteristic immunologic profile with higher PD-L1 expression in contrast to *WRN* wildtype which could impact response to immunotherapy (Zimmer et al., 2020).

Breast cancer cell lines have also demonstrated hypermethylation of *WRN*. Expression of *WRN* in a breast cancer cell line appears to inhibit tumor growth in athymic nude mice (Agrelo et al., 2006). In patients with breast cancer, methylation status of *WRN* was significantly correlated with expression (Li et al., 2016) and an independent study demonstrated that increased expression of *WRN* mRNA and protein were associated with improved overall survival (OS) and relapse-free survival (RFS) (Shamanna et al., 2016; Zhu et al., 2018).

Primary cervical cancer samples and cervical cancer cell lines were analyzed for *WRN* hypermethylation and found in 33.3% of patients and 33.3% of cancer cell lines (Masuda et al., 2012). Treatment with siRNA for *WRN* increased sensitivity of cancer cells to CPT-11, a topoisomerase I inhibitor. Decreased *WRN* mRNA expression negatively correlated with cervical cancer progression and WRN protein regulates the life cycle of viral carcinogen human papillomavirus 16 (HPV-16), linked with causing cervical and oropharyngeal cancers (James et al., 2020).

### 2.7 RECQL4

#### 2.7.1 Germline variants in *RECQL4* cause Rothmund-Thompson, RAPADILINO, and Baller-Gerold Syndromes

Rothmund-Thompson Syndrome (RTS) has two clinical subtypes, RTS I and RTS II. RTS I is characterized by poikiloderma, ectodermal dysplasia and juvenile cataracts, and a subset of these patients harbor variants in *ANAPC1* (Zirn et al., 2021). RTS II, on the other hand, is caused by autosomal recessive variants in the *RECQL4* gene located at the 8q24.3 locus (Wang et al., 2003; Mo et al., 2018; Lu et al., 2020) and is characterized by poikiloderma, congenital bone defects, and an increased risk of osteosarcoma and skin cancers, squamous and basal cell carcinomas (Lu et al., 2020). The developmental defects, including radial ray defects, classically seen in RTS II correlating with the presence of *RECQL4* variants suggest that RECQL4 likely plays a crucial role in both normal skeletal development and oncogenesis, in addition to other major signaling pathways such as Wnt, Hedgehog, and Notch (Wang et al., 2003; Hu et al., 2005; Glass and Karsenty, 2007; Cleton-Jansen et al., 2009; Kansara et al., 2009; Tao et al., 2010; Vijayakumar et al., 2011; Belyea et al., 2012; Tao et al., 2014; Lu et al., 2020).

A recent study examined the prevalence of cancer risk in patients carrying monoallelic pathogenic variants in the *RECQL4* gene (Martin-Giacalone et al., 2022). Despite some data suggesting that heterozygous germline variants in RECQL4 increase risk of osteosarcoma, in this analysis of an international registry of RTS II patients and their family members, investigators found that RTS II family members with heterozygous germline pathogenic variant in *RECQL4* did not have an increased risk of developing cancer, compared to the age-adjusted population estimate per the Surveillance, Epidemiology, and End Results (SEER) program (Maciaszek et al., 2019; Martin-Giacalone et al., 2022).

Other syndromes caused by variants in *RECQL4* include RAPADILINO syndrome and Baller-Gerold syndrome. In RAPADILINO syndrome, patients present with skeletal defects, including radial ray and limb deformities, small stature, palatal defects, and absent patella. These patients are susceptible to developing lymphoma and osteosarcoma (Wang et al., 2003). Patients with Baller-Gerold syndrome are typically characterized by craniosynostosis, poikiloderma, and radial ray defects. These patients are predisposed to developing lymphoma (Wang et al., 2003).

### 2.8 RECQL4 and Cancer

Patients with pathogenic variants in *RECQL4* are more susceptible to developing OS, thereby making RTS II a good model that can be used to understand OS and develop targeted therapy. Although OS risk is increased in patients with RTS, *RECQL4* variants have not been found in sporadic OS, suggesting that it may be targeted directly in germline but not somatic variants (Wang et al., 2003; Lu et al., 2020).

A previous study indicated that altered RecQ enzyme expression correlated with prognostic outcome in hematologic malignancies (Viziteu et al., 2016). Patients with AML with abnormal karyotype were found to have low RECQL4 and BLM expression, which correlated with adverse outcomes. On the other hand, overexpression of RECQL4 indicated a better prognosis. In multiple myeloma patients, overexpression of RECQL1, WRN, and RECQL4 were associated with poor prognosis (Viziteu et al., 2016).

Upregulated *RECQL4* expression has been found to correlate with increased tumor aggressiveness in human prostate cancer cells (Su et al., 2010; Mo et al., 2018). A study demonstrated that *RECQL4* inhibition in prostate cancer cells led to significant reduction in invasive growth *in vitro* and tumorigenic potential *in vivo* (Su et al., 2010; Mo et al., 2018). The *RECQL4* harboring chromosome region (8q24.3) became amplified, and the level of amplification corresponded with tumor aggressiveness. This correlation may be beneficial as a prognostic tool in metastatic prostate cancer (Mo et al., 2018).

Tumors with increased *RECQL4* expression may be resistant to radiation therapy (Jiang et al., 2009). After mitochondria in Raji cells were exposed to radiation, proteomic analysis revealed that RECQL4 along with GAPDH, MK167, and ATAD3B proteins can contribute as biomarkers for radiation resistance (Jiang et al., 2009). While it is unclear whether RECQL4 expression in cancer cells is related to radiation resistance, a preliminary study showed that RECQL4 inhibition enhanced radiation sensitivity of prostate cancer cells (Jiang et al., 2009; Mo et al., 2018).

Similar to prostate cancer cells, breast cancer cells show overexpression of *RECQL4* (Fang et al., 2013; Mo et al., 2018) A study demonstrated that the majority of the breast cancer cells exhibited gene amplification via increased 8q24 (Fang et al., 2013). Studies reported that *RECQL4* is not only a metastasis-promoting gene but also a prognostic indicator in breast cancer, as elevated levels of *RECQL4* gene amplification, mRNA, and protein demonstrated considerably increased tumor aggressiveness (Thomassen et al., 2009; Santarpia et al., 2013; Arora et al., 2016a; Mo et al., 2018; Zhu et al., 2018). This association between overexpression and cancer progression highlights the potential clinical benefit of targeting *RECQL4* in breast cancer.

A study analyzed five human lines with esophageal squamous cell carcinoma (ESCC) and found that *RECQL4* expression was significantly increased in tumor tissues in contrast to non-tumor tissues (Lyu et al., 2021). Additionally, *RECQL4* overexpression was associated with worse survival outcomes, including poor tumor differentiation, lymph node invasion, and metastatic disease. When *RECQL4* was depleted, the cells were arrested in G0/G1 phase, and cell senescence occurred. Depletion also led to enhanced DNA damage, production of reactive oxygen species and impaired DNA damage response via the phosphorylation or activation of the kinases ATM, ATR, CHK1, and CHK2 (Lyu et al., 2021).

When ovarian cancer tissues were examined, upregulation of *RECQL4* positively correlated with enhanced cell proliferation and invasion relating to potential worse survival (Guo et al., 2020). *RECQL4* knockout caused cell cycle arrest as well as apoptosis. RECQL4 depletion led to increased sensitivity of the ovarian cancer cells to cisplatin and olaparib, a PARP inhibitor, which suggests that *RECQL4* may be a critical component in the resistance of ovarian malignant cells to cisplatin. MAFB, involved in cell differentiation and oncogenesis, is a downstream effector of *RECQL4* and expression of *MAFB* was associated with *RECQL4* expression. *MAFB* silencing led to reduced cell viability, proliferation, and invasion. Furthermore, miR-10a-5p, a tumor suppressor, negatively regulates *RECQL4* expression, thus suggesting its role in *RECQL4* overexpression and, subsequently, oncogenic effect (Guo et al., 2020).

Similar to esophageal and ovarian cancers, *RECQL4* mRNA expression was also increased in human gastric cancer and hepatocellular carcinoma cells, correlating with poor prognosis. Gastric cancer cells with enhanced *RECQL4* expression correlated with more extensive invasion, as compared with normal gastric mucosa cells (Chen et al., 2018). Overexpression of *RECQL4* in more than half of the seven gastric cancer cell lines analyzed, were not only associated with poor prognosis but also with enhanced resistance to cisplatin via the downstream AKT-YB1-MDR1 signaling pathway (Mo et al., 2016). In the setting of elevated endogenous *RECQL4*, silencing of ectopic *RECQL4* in cisplatin-resistant gastric cancer cells led to reduced activation of this pathway and subsequent resensitization to cisplatin (Mo et al., 2016). In hepatocellular carcinoma cells, *RECQL4* overexpression positively correlated with significantly shorter disease-free survival (DFS) and overall survival (OS) times compared with tissue showing lower *RECQL4* expression (Li et al., 2018b). These cells demonstrated features of poor prognosis, including elevated a-fetoprotein (AFP) levels and higher staging (Li et al., 2018b).

Overexpression of *RECQL4* mRNA and protein levels were also associated with poor survival outcomes in glioblastoma, while silencing of the gene led to significant chemosensitivity. Absence of RECQL4 in glioma cell lines demonstrated increased sensitivity to temozolomide via increased apoptotic proteins (Krol et al., 2020). This data highlights that targeting *RECQL4* may potentially improve prognosis of a variety of cancers.

### 2.9 RECQL5

#### 2.9.1 Germline Variants in *RECQL5*

Although there are no known syndromes associated with germline variants in *RECQL5*, there are a few studies that suggest that *RECQL5* could be a cancer susceptibility gene. In a Spanish study of 700 families with breast and ovarian cancer who were negative for variants in *BRCA1/2*, there were deleterious or likely deleterious variants in *RECQL5*, which was enriched compared to controls (Tavera-Tapia et al., 2019). Polymorphisms in *RECQL5* were associated with breast cancer, osteosarcoma, and laryngeal in a Chinese population (He et al., 2014; Qi and Zhou, 2014; Zhi et al., 2014).

### 2.10 RECQL5 and Cancer

RECQL5 deficiency is associated with genomic instability and thought to lead to cancer. *RECQL5* is overexpressed in human urothelial carcinoma of the bladder (UCC) tissue compared to control normal bladder tissue and is associated with negative outcomes (Patterson et al., 2016). *RECQL5* depletion in both UCC and normal bladder cells cause a significant decrease in cell survival in only the malignant cells. This differential effect of RECQL5 depletion on UCC cells compared to normal, suggests a role for RECQL5 targeted therapy (Patterson et al., 2016).

While overexpression of *RECQL5* in UCC led to poor prognosis, low expression in human gastric carcinoma (GC) samples correlated with worse overall survival (Lin et al., 2020). RECQL5 may be a prognostic indicator in GC, particularly relating to extent of disease invasion and aggressive histology.

Increased *RECQL5* mRNA expression was observed in breast cancer cells and associated with poor prognosis (Arora et al., 2016b). High *RECQL5* levels correlated with worse phenotypes, including high histological grade, increased *HER2* expression, and ER positivity. High RECQL5 protein expression combined with low RAD51 nuclear protein levels also correlated with worse survival outcomes (Arora et al., 2016b).

In the previously mentioned study investigating altered RecQ expression and prognostic value, overexpression of *BLM*, *RECQL1*, and *RECQL5* in AML patients with normal karyotype were associated with poor prognosis (Viziteu et al., 2016). *RECQL5* overexpression was also found in several other hematologic malignancies, such as diffuse large B cell lymphoma and chronic lymphocytic leukemia, correlated with poor outcomes (Viziteu et al., 2016).

Overexpression of *RECQL5* has been found in two subtypes of non-small cell lung cancer (NSCLC), lung adenocarcinoma (LUAD) and lung squamous carcinoma (LUSC), along with NSCLC cell lines (Xia et al., 2021). Upon further analysis, it was found that *RECQL5* depletion led to not only inhibition of invasion and migration of NSCLC cells but also suppression of lung metastasis. It also inhibited epithelial-mesenchymal transition (EMT), which is a function of metastasis by malignant cells (Valastyan and Weinberg, 2011; Xia et al., 2021).

## 3 Exploiting RecQ Helicases for Therapeutic Opportunity

To our knowledge, there are no approved or known clinical trials for therapies that directly target RecQ helicases (Futami and Furuichi, 2014; Hengel et al., 2017; Sommers et al., 2019). However, there are ongoing efforts to identify small-molecule inhibitors against RECQL1, BLM, and WRN (Futami and Furuichi, 2014; Hengel et al., 2017; Sommers et al., 2019). As the structure and biochemical interactions of these current biomolecular small-molecule inhibitors are further studied, a deeper understanding of their inhibitory effects in DNA repair pathways may lead to the development of successful targeted therapies.

Most traditional chemotherapy and radiation approaches to treat cancer cause DSBs or other DNA lesions that lead to subsequent cell death. When certain genes involved in DNA repair are altered through mutations or epigenetic silencing, cancer cells may rely on other repair pathways for survival. Synthetic lethality is a model where inhibition of two or more involved pathways can trigger cell death. For example, loss of BRCA1 or BRCA2 results in decreased HR, forcing cancer cells to utilize Poly (ADP-ribose) polymerases (PARP), an enzyme that facilitates repair of single-strand breaks and BER (Ame et al., 2004). When PARP is suppressed and single-strand breaks accumulate, stalled replication forks degrade into DSBs, which are then not able to be repaired in the setting of BRCA-deficient cancer cells. Thus, this synthetic lethality model has led to the development of multiple PARP inhibitors which are now approved to treat several different cancers, most effective in patients with germline BRCA alterations (Bryant et al., 2005; Farmer et al., 2005; Audeh et al., 2010; Tutt et al., 2010; Gelmon et al., 2011; Kaye et al., 2012; Mateo et al., 2016; de Bono et al., 2017).

### 3.1 RECQL1

Similar to PARP inhibitors, topoisomerase I (TOP1) inhibitors, including camptothecin (CPT), prevent DNA repair through the development of replication-mediated DSBs, and are used for the treatment of several metastatic cancers (Fox et al., 2003; Pommier et al., 2003; Pommier et al., 2006; Berti et al., 2013). TOP1 inhibitors trap the TOP1 cleavage complex, a catalytic intermediate consisting of TOP1 covalently linked to supercoiled DNA, which are then transformed into replication-induced DSBs. Additionally, low doses of TOP1 inhibitors stall replication, further impairing DNA repair (Fox et al., 2003; Pommier et al., 2003; Pommier et al., 2006; Berti et al., 2013). One of the unique features of RECQL1 compared to the rest of the RecQ enzyme family is that it is able to drive recovery of DNA replication forks after they undergo structural reversal that results from replication stress secondary to TOP1 inhibition. One study showed that PARP1 is required to regulate RECQL1 activity in this state and it, therefore, stabilizes regressed forks (Berti et al., 2013). PARP1 suppression combined with TOP1 inhibition, even at low doses, can lead to replication-mediation DSBs (Fox et al., 2003; Pommier et al., 2003; Pommier et al., 2006; Berti et al., 2013). This may imply that when both PARP1 and TOP1 are inhibited in cells depleted of homologous recombination, further inhibition by RECQL1 may potentiate the outcome by TOP1 inhibition and significantly impair DNA repair (Pommier et al., 2006).

As previously mentioned, siRNA silencing of *RECQL1* has been shown to kill various HCC, ovarian cancer cell lines, and hypopharyngeal carcinoma and have *in vivo* activity in mice (Futami et al., 2010; Arai et al., 2011; Sanada et al., 2013). This approach could be effective against other cancers as well. The mechanism of anticancer activity was via mitotic catastrophe, which was even enhanced with the addition of chemotherapeutic agents (Futami et al., 2008b; Futami and Furuichi, 2014).

The study by Viziteu et al. demonstrated that *RECQL1* depletion sensitizes multiple myeloma cells to PARPi-induced apoptosis (Viziteu et al., 2016). The data suggests that future therapeutic targets against multiple myeloma can potentially combine DNA methyltransferase inhibitors (DNMTi), PARPi, and inhibitors against RECQL1 helicase to both downregulate RECQL1 activity in replication stress and minimize resistance to chemotherapeutic agents (Viziteu et al., 2017).

### 3.2 BLM

The first small-molecule inhibitor against BLM is ML216 (1-(4-fluoro-3-trifluoromethyl)phenyl)-3-(5-(pyridine-4-yl)-1,3,4-thiadiazol-2-yl) urea). ML216 and its derivatives inhibit BLM by interfering in the BLM-ssDNA network and impairing cellular proliferation though they do not appear to be highly specific against BLM (Hengel et al., 2017). RNAi-mediated shRNA gene silencing allowed for depletion of *BLM*, which led to inhibition of specific osteosarcoma cells, implicating its potential use in osteosarcoma (Mao et al., 2010; Futami and Furuichi, 2014).

One study demonstrated that BLM enhances c-Myc turnover by interacting with ligase Fbw7 and promoting its degradation (Chandra et al., 2013). In a mouse xenograft model, BLM inhibits c-Myc dependent colon cancer initiation (Chandra et al., 2013). This opens the possibility of additional pathways that could be attractive therapeutic targets.

### 3.3 WRN

WRN has been identified as being an attractive synthetic lethal target in the setting of microsatellite unstable cancers (Chan et al., 2019; Kategaya et al., 2019; Lieb et al., 2019). Microsatellite instability (MSI) resulting from DNA mismatch repair deficiency leads to several different cancers, including gastrointestinal cancers and gynecologic cancers (Gurin et al., 1999; Boland and Goel, 2010). Decreased *WRN* causes catastrophic DNA damage in MSI cells, leading to cell cycle arrest and apoptosis (Chan et al., 2019; Kategaya et al., 2019; Lieb et al., 2019). One mechanism for this synthetic lethality is through accumulation of DSBs at TA-dinucleotide repeats (van Wietmarschen et al., 2020). These TA-dinucleotide repeats are prone to stalls in replication forks, which requires unwinding by the WRN helicase. Without WRN, these TA-dinucleotide repeats are cleaved by MUS81 nuclease, leading to massive genomic instability (van Wietmarschen et al., 2020). This could form the basis of WRN-directed cancer therapy.

With regard to ionizing radiation therapy, WRN-deficient cells are dependent on CHK1 mediated homologous recombination repair (Gupta et al., 2021). It has been shown that the CHK1-p38-MAPK pathway is important in the setting of WRN-deficient cells. Thus, targeting of CHK1 in the setting of WRN deficiency, results in radiosensitivity, as shown in melanoma tumors *in vivo* (Gupta et al., 2021).

NSC 19630 (1-(propoxymethyl)-maleimide) and its derivative NSC 617145 are the first human DNA small-molecule inhibitors against WRN helicase. Studies have shown that these inhibitors of WRN helicase trap WRN on DNA as well as lead to the accumulation of stalled replication forks and apoptosis, compromising cellular proliferation (Hengel et al., 2017). Treatment with NSC 19630 was able to induce apoptosis in human T-cell leukemia virus type 1 (HTLV-1)-transformed T-cell leukemia/lymphoma-derived cell lines (Moles et al., 2016). This data, in addition to the prevalence of hematologic neoplasms in Werner Syndrome patients, suggests the beneficial impact of inhibiting WRN helicase in leukemia cells in particular.

A large-scale high-throughput screen of about 350,000 small molecules identified other small-molecule inhibitors of WRN helicase (Sommers et al., 2019). NCGC00029283-03 and NCGC00063279-03 are partially reversible inhibitors, which make them suitable drug candidates as irreversible inhibitors may function nonspecifically (Sommers et al., 2019). Both showed favorable chemical properties and biological activity unlike other identified molecules, and highlight the potential role of WRN helicase inhibition (Sommers et al., 2019).

### 3.4 RECQL4

Cancer stem cells possess high proliferative capacity and resistance to DNA damage and cell death via constitutive checkpoint inhibitor and repair mechanisms (Skvortsova et al., 2015; Abad et al., 2020; Balajee, 2021). The authors of a review explained that *RECQL4* expression may be important for stemness, as demonstrated by a positive association between *RECQL4* and some stem cell markers, including Myc and CD133 (Balajee, 2021). They proposed that since *RECQL4* expression may contribute to the unlimited proliferative potential and survival of neoplastic stem cells, *RECQL4* is a potential target that would address these DNA damage-resistant cells. Studies show higher *RECQL4* expression was observed in glioblastoma stem cells, whose development was impaired upon RECQL4 inhibition (Krol et al., 2020; Balajee, 2021). Additionally, *RECQL4* expression significantly correlates with its downstream target, MAFB, a transcription factor whose inhibition affects aggressiveness of ovarian cancer cells as well as osteosarcoma stem cells (Chen et al., 2020; Guo et al., 2020; Balajee, 2021).

Previous studies also found that the antibiotic heliquinomycin suppressed the replication effects of many DNA helicases, including RECQL4 (Sugiyama et al., 2012; Balajee, 2021). Future therapies can potentially use compounds like heliquinomycin to inhibit RECQL4-induced replication of cancer cells.

### 3.5 RECQL5

Investigators developed a small-molecule inhibitor, compound 4a, which is a 1,3,4-oxadiazole derivative that specifically targeted RECQL5-positive breast cancers (Chakraborty et al., 2021). By securing the RECQL5-RAD51 interaction, this compound suppressed homologous recombinatorial repair, induced DNA double-stranded breaks, and inhibited tumorigenesis. This process led to preferential killing of RECQL5-expressing cancer cells. In addition to targeting RECQL5 specifically, the compound was also designed to evade neoadjuvant/adjuvant mediated chemoresistance often encountered in breast carcinoma (Chakraborty et al., 2021).

Colorectal carcinomas are often treated with camptothecins (CPTs), including irinotecan and topotecan, which are type I topoisomerase inhibitors. However, tumors may develop resistance against these therapies (Strumberg et al., 2000; Furuta et al., 2003; Wang et al., 2011b). A study investigated whether *RECQL5* plays a role in CPT resistance in colorectal carcinoma. When two colorectal cancer cell lines were depleted of *RECQL5*, CPT sensitivity was remarkedly increased as compared to parental lines containing *RECQL5*. Additionally, CPTs successfully treated xenograft tumors derived from one of the *RECQL5*-deficient neoplastic cell lines though not cells consisting of *RECQL5*. This data suggested that *RECQL5* likely contributes to CPT resistance in colorectal carcinoma and may be used as directed targeted therapy to treat colorectal malignant disease (Wang et al., 2011b).

The PI3K/Akt signaling pathway is one studied way in which neoplasms can proliferate and induce EMT as well as cause resistance to platinum-based treatments (Valastyan and Weinberg, 2011; Xia et al., 2021). When Akt inhibitor LY294002 was added, the effects of *RECQL5* overexpression were reversed, overall leading to reduced proliferation and metastasis via EMT. *RECQL5* knockdown also enhanced apoptosis of cisplatin-resistant cells upon cisplatin treatment (Xia et al., 2021). This study also suggests the therapeutic implications of targeting *RECQL5* in non-small cell lung cancer.

## 4 Conclusion

RecQ helicases are a highly conserved family of enzymes involved in several pathways to maintain genomic stability. Of the five different proteins in humans, RECQL1, BLM, WRN, RECL4, and RECQL5, three are associated with germline alterations associated with an increased risk of cancer. This review has focused on somatic alterations and how loss of RecQ helicase function and upregulated expression have been shown to correlate with tumorigenesis. We also review novel strategies in which RecQ helicases can be exploited for anti-cancer therapies either through direct targeting via small molecules or synthetic lethality. Further studies are needed to better understand the mechanisms of the role of RecQ helicases in cancer which will have broad implications beyond cancer, including in the fields of DNA repair, cancer susceptibility, and aging.
